# The prognostic implications and tumor-suppressive functions of CYR61 in estrogen receptor-positive breast cancer

**DOI:** 10.3389/fimmu.2023.1308807

**Published:** 2024-01-08

**Authors:** Cheng Zhang, Zhihua Li, Kaiheng Hu, Yifei Ren, Haoran Zhang, Yuankang Zhao, Wenjing Wei, Shuo Tu, Xiaohua Yan

**Affiliations:** ^1^ The MOE Basic Research and Innovation Center for the Targeted Therapeutics of Solid Tumors, School of Basic Medical Sciences, Jiangxi Medical College, Nanchang University, Nanchang, China; ^2^ Department of GCP, The First Affiliated Hospital, Jiangxi Medical College, Nanchang University, Nanchang, China; ^3^ Department of Breast Surgery of Third Hospital of Nanchang and Key Laboratory of Breast Diseases of Jiangxi, Nanchang, China; ^4^ Queen Mary School, Jiangxi Medical College, Nanchang University, Nanchang, China

**Keywords:** Cyr61, ER-positive breast cancer, tumor immune microenvironment (TIME), immune cell infiltration, cancer cell proliferation

## Abstract

Due to the therapeutic resistance of endocrine therapy and the limited efficacy of immune checkpoint inhibitors in estrogen receptor (ER)-positive breast cancer (BRCA), there is an urgent need to develop novel prognostic markers and understand the regulation of the tumor immune microenvironment (TIME). As a matricellular protein, CYR61 has been shown to either promote or suppress cancer progression depending on cancer types. However, how CYR61 functions in ER-positive BRCA remains elusive. In this study, we comprehensively analyzed the expression of CYR61 in BRCA based on the TCGA and METABRIC databases. Our findings showed that the expression of CYR61 is downregulated in different subtypes of BRCA, which is associated with elevated promoter methylation levels and predicts bad clinical outcomes. By comparing the high or low CYR61 expression groups of ER-positive BRCA patients, we found that CYR61 is intimately linked to the expression of genes involved in tumor-suppressive pathways, such as the TGF-β and TNF signaling pathways, and genes related to cytokine-receptor interaction that may regulate cancer immunity. Moreover, reduced CYR61 expression is associated with an altered TIME that favors cancer progression. Finally, experimental analyses ascertained that CYR61 is downregulated in clinical BRCA tissues compared to matched normal breast tissues. Furthermore, CYR61 is able to impede the proliferation and colony formation of ER-positive BRCA cells. In summary, our study reveals that CYR61 could serve as a novel prognostic marker for ER-positive BRCA, and function as an inhibitor of cancer progression by both acting on cancer cells and remodeling the TIME.

## 1 Introduction

Breast cancer (BRCA) accounts for one-third of all female patient tumor occurrences, posing a serious threat to women’s lives and health worldwide (1). Based on disparities in immunohistochemical markers, BRCA can be further classified into estrogen receptor (ER)-positive, progesterone receptor (PR)-positive, HER2-positive and triple-negative (ER-/PR-/HER2-) subtypes (2, 3). The development of both normal mammary tissue and ER-positive BRCA depend heavily on estrogen and its receptors (4). Although endocrine therapy has been shown effective in hormone-positive BRCA, drug resistance and cancer relapse remain significant challenges (5, 6). With our increasing understanding of the tumor immune microenvironment (TIME), various immunotherapies, such as immune checkpoint inhibitors (ICIs), have been developed and considered promising for triple-negative breast cancer (TNBC) (7). However, the efficacy of immunotherapy in ER-positive BRCA seems to be less satisfied (3, 5). Therefore, there is an urgent need to develop new prognostic molecular makers and to understand the regulation of the immune microenvironment of ER-positive BRCA.

The cysteine-rich angiogenesis inducer 61 (CYR61, also known as CCN1) is an extracellular secretory matrix protein. CYR61 has been appreciated for playing an important role in various biological processes, such as angiogenesis, tissue repair, inflammation response, cell migration and invasion (8, 9). The dysregulation of CYR61 has been found in different human diseases including cancer (9, 10). Previous studies have found that CYR61 expression is upregulated in gliomas and pancreatic carcinoma, whereas downregulated in prostate carcinoma and non-small cell lung carcinoma (10–12). Interestingly, CYR61 has been reported to promote or suppress cancer progression, depending on the type and stage of cancer (9, 10). As a typical transcriptional target of the YAP/TEAD signaling pathway, CYR61 was reported to be negatively correlated with the expression level of ESR1 that encodes estrogen receptor alpha, in BRCA (13, 14). However, it remains to be further elucidated whether the expression of CYR61 may serve as a prognostic marker for BRCA and whether CYR61 may regulate the TIME.

In this study, through comprehensive analyses of different cancer databases, we found that CYR61 expression was significantly downregulated in different subtypes of BRCA, including ER-positive BRCA. Reduced CYR61 expression was associated with increasing levels of its promoter methylation, and predicted reduced survival rates in cancer patients. Intriguingly, analyses of ER-positive BRCA patients in The Cancer Genome Atlas Program (TCGA) database also indicated that CYR61 was associated with some tumor-suppressor pathways and remodeling of the TIME. In addition, experimental studies verified that CYR61 was downregulated in clinical breast cancer tissues compared to matched normal tissues, and demonstrated that CYR61 can inhibit the proliferation and growth of ER-positive BRCA cells. These results reveal that CYR61 exerts an inhibitory role in ER-positive BRCA by regulating cancer cell proliferation and the TIME remodeling, and may serve as an independent prognostic marker.

## 2 Materials and methods

### 2.1 Acquisition and processing of data

The mRNA matrix, methylation matrix, and survival data of BRCA patients were obtained from TCGA. These datasets were coupled with transcriptional data from the TCGA-pancancer cohorts and the Genotype-Tissue Expression (GTEx) database. Access to these datasets was facilitated through UCSC Xena (http://xena.ucsc.edu/). The relevant clinical data of these BRCA patients were acquired utilizing the R package “TCGAbiolinks”. The mRNA matrix, methylation matrix, and relevant survival data of the Molecular Taxonomy of Breast Cancer International Consortium (METABRIC) cohorts were obtained from cBioPortal (https://www.cbioportal.org/), a platform dedicated to multi-omics tumor genetics analysis. Then, all attained transcriptional data were transformed into the log_2_(TPM+1) format, an essential step preparing them for subsequent statistical analyses. The transformation of probe identifiers was executed utilizing the R package “tinyarray”.

### 2.2 Inclusion criteria of patients and samples from TCGA and METABRIC databases

Breast cancer patients meeting specific eligibility criteria were selected for the subsequent data analyses. First, breast tissue samples with available mRNA expression data were considered. Second, one sample per patient was retained, with preference shown for frozen samples. Lastly, TCGA patients with both overall survival (OS) and disease-specific survival (DSS) data, and METABRIC patients with both OS and relapse-free survival (RFS) data were selected for the analyses.

### 2.3 Gene expression analysis by cancer databases

To clarify the correlation between CYR61 expression level and BRCA, boxplots based on CYR61 expression were plotted using the TCGA-pancancer cohorts and GTEx datasets. Additionally, CYR61 mRNA expression boxplots were plotted for normal breast tissue, ER-positive and/or PR-positive BRCA samples from the TCGA and METABRIC cohorts. Paired boxplots were also generated based on matched tumor samples and normal tissue from TCGA. BRCA samples were grouped based on various clinical features (PAM50, Stage, T stage, M stage, N stage), and boxplots were sequentially drawn according to CYR61 expression within each group.

### 2.4 DNA methylation analysis

All probe numbers corresponding to the CYR61 promoter were obtained from Mexpress (https://mexpress.be/). The final β value of CYR61 for each sample was calculated as the average of β values from the relevant probes. Boxplots were plotted based on the β values of CYR61 for each sample. Dotted plots were generated based on both mRNA and β values for each sample. Similar to expression analysis, BRCA samples were divided into different groups based on clinical features (PAM50, Stage, T stage, M stage, N stage), and boxplots were drawn based on the degree of CYR61 methylation in each group.

### 2.5 Prognostic analysis

Kaplan-Meier curve analyses were performed using BRCA and ER-positive BRCA groups in TCGA BRCA cohorts and METABRIC cohorts. The optimal cutoff point was determined using the “surv_cutpoint” function from the R package “survminer”, considering CYR61 expression. The log-rank P value was computed.

### 2.6 Pathway enrichment analysis

Enrichment analysis was carried out based on CYR61 expression levels in pre- and post-10% ER-positive BRCA samples. Genes expressed in at least 50% samples with a count of more than 30 were subjected to differential expression analysis with a threshold of |logFC| >1 and p <0.05 for the two groups. The resulting differential genes (DEGs) were enriched using the “clusterProfiler” package for gene ontology (GO) and KEGG analyses. Gene Set Enrichment Analysis (GSEA) was utilized to further analyze the relationships between CYR61 expression and key pathways.

### 2.7 Immune cell infiltration analysis

Immune cell infiltration was predicted using the R package “CIBERSORT” based on the sample’s expression matrix. CIBERSORT deconvolution algorithm was used to analyze the relative fractions of 22 infiltrating immune cell types in CYR61 samples from TCGA datasets. Samples were divided into high-expression and low-expression groups according to medium CYR61 expression. Violin plots were generated to illustrate immune cell infiltration differences. For immune cells showing significant differences in infiltration, dot plots were created to visualize the correlation of CYR61 mRNA expression with cell infiltration. To further explore the association between CYR61 and immune infiltration, the “ESTIMATE” package was utilized to estimate the scores (ESTIMSTE score, stromal score, immune score) for each sample. The correlation between these scores and CYR61 expression was displayed through heatmaps.

### 2.8 Cell culture

Human ER-positive breast cancer cell line MCF-7 was obtained from Cell Bank/Stem Cell Bank, Chinese Academy of Sciences. MCF-7 was cultured in RPMI 1640 medium (Solarbio, Beijing), supplemented with 10% fetal bovine serum (FBS, Gibco, USA) in a humidified incubator at 37°C in 5% CO2 atmosphere.

### 2.9 Gene silencing and reagents

The non-specific control siRNA (NC) and those targeting human CYR61 were purchased from RiboBio (China). siRNAs were transfected with the siTranTM siRNA transfection reagent (OriGene). The sequences of siRNAs used in this study were as follow: CYR61 siRNA #1, 5’-CCACACGAGTTACCAATGA-3’; CYR61 siRNA #2, 5’-GAACCAGTCAGGTTTACTT-3’. Recombinant human CYR61 (CB98) peptides was purchased from Novoprotein (Shanghai).

### 2.10 Collection of clinic breast cancer samples

A total of seven ER-positive BRCA samples and the adjacent matched normal tissues were excised from inpatients at the department of breast surgery of Third Hospital of Nanchang. The tumor tissues were stored in liquid nitrogen after excising from cancer patients. Informed consent was obtained from all individuals participating in this study. The usage of clinical sections was consented by the Medical Ethics Committee of Third Hospital of Nanchang. The processes of clinical sample collection and usage were in strict accordance with the guideline. All procedures performed in this study were in accordance with the ethical standards of the Medical Ethics Committee of Third Hospital of Nanchang.

### 2.11 Lentivirus production and stable cell line establishment

To produce defective lentivirus, HEK293FT cells were transfected with the empty lentivirus vector pL6.3-CMV-GFP-IRES-MCS or the CYR61-expressing derivative, along with the package plasmids pCMVΔ8.9 and VSVG. The culture supernatants were collected at 48 h post-transfection, and the viral particles were concentrated by centrifugation. To establish CYR61- or vector-expressing stable cell lines, MCF-7 cells were infected with lentivirus particles at a multiplicity of infection (MOI) of 50 pfu per cell. At 48 h post-infection, the cells were washed with PBS, complemented with fresh growth medium containing 1 μg/mL of blasticidin. The drug-resistant cells were pooled as stable cells, which were further maintained with blasticidin-containing growth medium.

### 2.12 RNA purification and real-time quantitative PCR

RNA purification and reverse transcription were performed as previously described (15). Briefly, total RNA was extracted from ER-positive BRCA tissues and matched paracancer tissues, from 7 patients undergoing surgery in Third Hospital of Nanchang, then reverse-transcribed into cDNA. Next, the relative mRNA expression of genes was normalized to that of GAPDH, and the fold change was evaluated using the 2−ΔΔCT method. The primer sequences used for q-PCR were obtained from Sangon Biotech (Shanghai, China): CYR61, forward 5’-GGTCAAAGTTACCGGGCAGT-3’ and reverse 5’-GGAGGCATCGAATCCCAGC-3’; GAPDH forward 5’-ACAACTTTGGTATCGTGGAAGG-3’ and reverse 5’-GCCATCACGCCACAGTTTC-3’.

### 2.13 Western blotting

For breast tissues, after homogenization, the samples were lysed with RIPA lysis buffer (R0020, Solarbio, Beijing), and the protein lysates were obtained after high-speed centrifugation. Likewise, cells were lysed with RIPA lysis buffer as above. Lysates were then separated by 10% SDS-PAGE and transferred to PVDF membranes to incubate with primary antibodies, including CYR61 (39382, CST, USA) and Tubulin (3873, CST, USA), followed by further incubation with the corresponding secondary antibodies. The bands on the membranes were finally visualized using the enhanced chemiluminescence (ECL) substrate (ECL-P-500, Yanxi Biotech, Shanghai) using the Tanon (Tanon 5200, Shanghai) Automatic Chemiluminescence Imaging System.

### 2.14 MTT and CCK8 assays

1×10^3^ viable cells were cultivated per well on 96-well plates in a final volume of 100 μl DMEM containing 10% FBS. For MTT assays, after 1-4 days of incubation, each sample was added with 20 μL per well of MTT solution (M8180, Solarbio, Beijing) and incubated at 37^°^C for 4 h. After aspiration of the supernatants, cells were incubated with 150 μL of DMSO (Solarbio, Beijing) at 37^°^C for 30 min. The absorbance was measured at 490 nm in a SpectraMaxR ParadigmR microplate reader (Molecular Decices, USA). For CCK8 assays, after 1-4 days of incubation, each sample was added with 10 μL per well of CCK8 solution (CA1210, Solarbio, Beijing) and incubated at 37^°^C for 2 h. The absorbance was detected at 450 nm in a SpectraMaxR ParadigmR microplate reader (Molecular Decices, USA).

### 2.15 Colony formation assay

Clonogenic growth of MCF-7 cells was determined by plating 1×10^3^ cells in 3 mL of growth medium in 6-well plates, and each sample was performed in triplicate. After incubation for 10-15 days, cells were fixed with methanol and stained with crystal violet solution, then the colony cells were imaged, and colony numbers were calculated by the ImageJ software.

### 2.16 Statistical analysis

Statistical analyses were performed using R (version 4.2.2) and GraphPad Prismversion 8.0). The significance between the means was calculated using wilcoxon rank-sum test, whereas paired t-test was conducted to compare paired tissue samples. Each experiment was performed at least in triplicate, and the values were presented as mean ± SD. The Kaplan-Meiermethod and log-rank test were used for survival analysis of cancer patients.

## 3 Results

### 3.1 Analysis of CYR61 gene expression in pan-cancer and breast cancer

**Figure 1:**
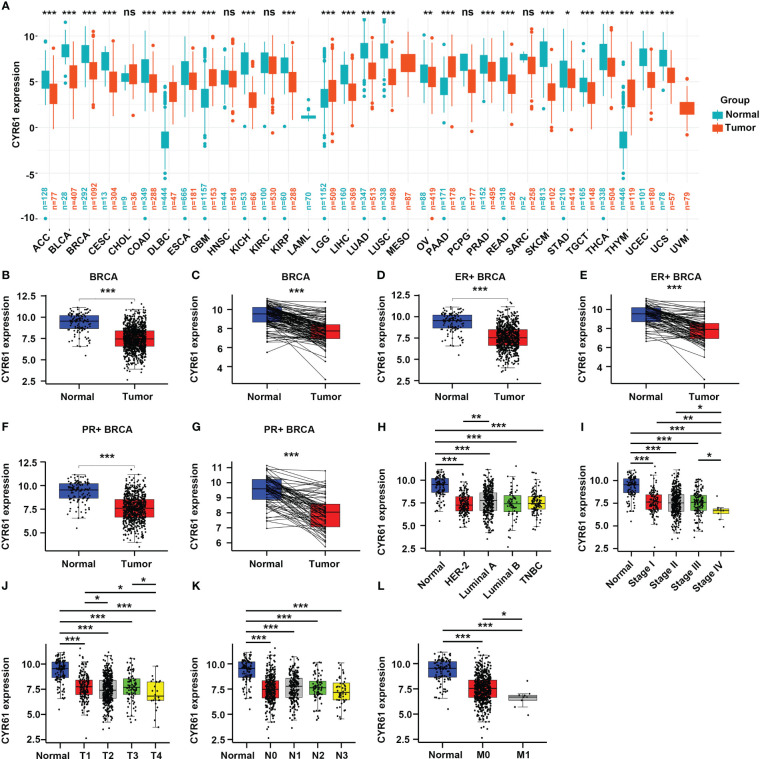
CYR61 expression is downregulated in BRCA than in the normal counterparts. **(A)** Comparation of the CYR61 mRNA expression levels between tumor samples and normal samples in different subtypes of BRCA based on TCGA and GTNx databases. **(B, C)** Comparation of the CYR61 mRNA expression levels between all available BRCA samples and normal breast samples **(B)**, or between matched BRCA samples and normal breast samples **(C)** in the TCGA database. **(D, E)** Comparation of the CYR61 mRNA expression levels between all ER+ BRCA samples and normal breast samples **(D)**, or between paired ER+ BRCA samples and normal samples **(E)** in the TCGA database. **(F, G)** Comparation of the CYR61 mRNA expression levels between all PR+ BRCA samples and normal breast samples **(F)**, or between matched PR+ BRCA samples and normal samples **(G)** in the TCGA database. **(H–L)** Comparation of the CYR61 mRNA expression levels in normal breast samples and in BRCA samples of different PAM50 status **(H)**, cancer stages (stage I, II, III and IV) **(I)**, primary tumor site stages (T1, T2, T3 and T4) **(J)**, lymph node stages (N0, N1, N2 and N3) **(K)** or metastatic spread stages (M0 and M1) **(L)** in the TCGA database. *p<0.05, **p<0.01, ***p<0.001. ns, no significance.

To investigate the roles of CYR61 in BRCA, we first explored its expression levels in pan-cancer based on public TCGA and GTEx databases. As a result, CYR61 was downregulated in most of the cancer types, such as BRCA, adrenocortical carcinoma (ACC), bladder urothelial carcinoma (BLCA), kidney chromophobe (KICH) and liver hepatocellular carcinoma (LIHC), amongst others (**Figure 1A**).

Then we specifically studied the expression of CYR61 in BRCA tissues based on the TCGA database. CYR61 expression level was downregulated in BRCA tissues compared to normal breast tissues, as assessed in all available cancer and normal tissue samples, as well as in paired tissues (**Figures 1B, C**). The expression of CYR61 was also reduced in ER-positive or PR-positive BRCA tissues when compared to normal breast tissues (**Figures 1D–G**). Similar results were obtained when the HER2-positive, luminal subtypes or triple-negative breast cancer were analyzed (**Figure 1H**). Furthermore, we found that the expression level of CYR61 was downregulated in different stages of BRCA tissues compared with normal tissues (**Figures 1I–L**).

### 3.2 Enhanced promoter methylation levels correlate with downregulated expression levels of CYR61 in breast cancer

**Figure 2:**
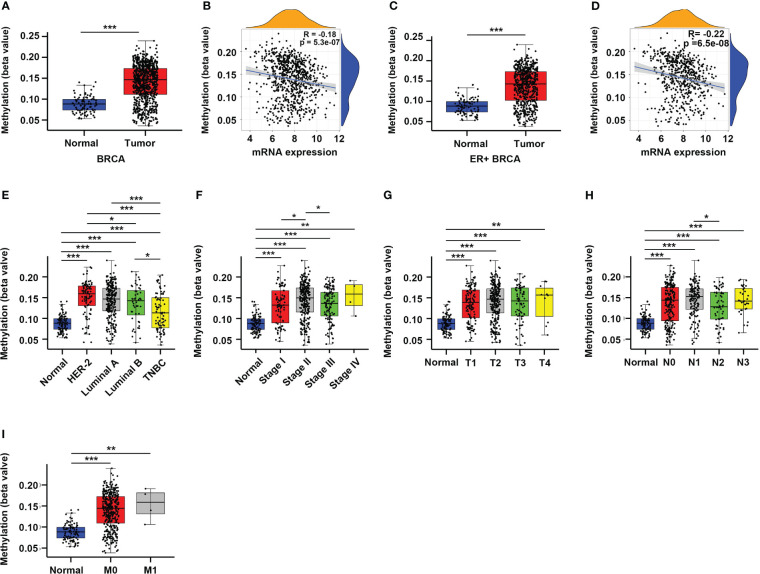
Enhanced promoter methylation level correlates with reduced CYR61 expression in BRCA patients. **(A, C)** Comparation of CYR61 gene methylation levels in normal breast samples with that in all BRCA samples **(A)** or in ER+ BRCA samples **(C)**. **(B, D)** Correlations of the methylation levels of CYR61 gene with its expression levels in all BRCA samples **(B)** or in ER+ BRCA samples **(D)**. **(E–I)** Comparation of the CYR61 gene methylation level in normal breast samples with that in BRCA samples of different PAM50 status **(E)**, cancer stages **(F)**, primary tumor site stages **(G)**, lymph node stages **(H)** or metastatic spread stages **(I)** in the TCGA database. *p<0.05, **p<0.01, ***p<0.001.

DNA methylation plays an important role in regulating gene expression, especially those occur in the gene promoter regions (6, 16). Therefore, we sought to explore whether the altered expression of CYR61 in BRCA is related to its promoter methylation level. Indeed, analyses of the TCGA datasets showed that CYR61 promoter methylation levels were significantly upregulated in BRCA tissues compared to normal breast tissues (**Figure 2A**), which closely correlated with the decreased expression level of CYR61 in BRCA (**Figure 2B**). Similar results were obtained in ER-positive BRCA (**Figures 2C, D**). In addition, the methylation level of the CYR61 promoter was also greatly upregulated in different subtypes or developmental stages of BRCA than those in normal breast tissue (**Figures 2E–I**). These observations were also consistent with the reduced CYR61 expression levels in BRCA (**Figures 1F–M**).

### 3.3 The prognostic value of CYR61 expression in breast cancer

**Figure 3:**
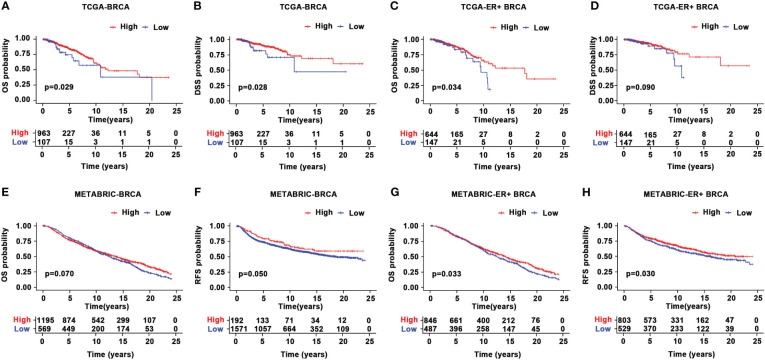
Prognostic value of CYR61 expression in BRCA. **(A, B)** Kaplan-Meier survival curve analyses illustrating the OS **(A)** and DSS **(B)** rates of high or low CYR61-expressing BRCA patients in the TCGA database. **(C, D)** Kaplan-Meier survival curve analyses illustrating the OS **(C)** or DSS **(D)** rates of high or low CYR61-expressing ER-positive BRCA patients in the TCGA database. **(E, F)** Kaplan-Meier survival curve analyses illustrating the OS **(E)** or RFS **(F)** rates of high or low CYR61-expressing BRCA patients in the METABRIC database. **(G, H)** Kaplan-Meier survival curve analyses illustrating the OS **(E)** or RFS **(F)** rates of high or low CYR61-expressing ER-positive BRCA patients in the METABRIC database.

Given the aberrant expression of CYR61 in BRCA, we analyzed the relationships between CYR61 expression levels and patient prognosis. Survival curve analyses based on the TCGA datasets indicated that lower CYR61 expression levels were associated with shorter overall survival (OS) and disease-specific survival (DSS) rates, in all BRCA patients (**Figures 3A, B**) or ER-positive BRCA patients (**Figures 3C, D**). Moreover, these results were confirmed by survival curve analyses based on the METABRIC data, assessed either in all BRCA patients (**Figures 3E, F**) or ER-positive BRCA patients (**Figures 3G, H**).

### 3.4 CYR61 is linked to remodeling of TIME in breast cancer

**Figure 4:**
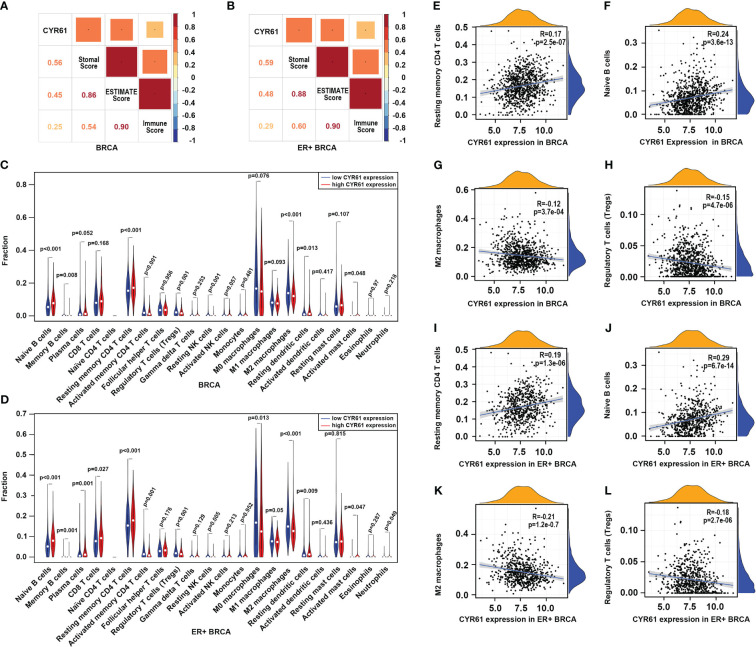
Downregulation of CYR61 is associated with a reduced antitumor immunity in the TME of BRCA patients. **(A, B)** Correlations of CYR61 expression level with the ESTIMATE scores assessed with BRCA samples **(A)** or ER+ BRCA samples **(B)** in the TCGA database. **(C, D)** Violin graphs showing the different immune cell infiltrations between low and high CYR61-expressing BRCA groups **(C)** or ER+ BRCA groups **(D)** in the TCGA database. Different groups were divided by the medium expression level of CYR61. The white dots indicate the medium levels of immune cell infiltrations. **(E–H)** Scatter plots showing the correlations of CYR61 expression with infiltrated resting memory CD4 T cells **(E)**, naive B cells **(F)**, M2 type macrophages **(G)** and Treg cells **(H)** in BRCA samples in the TCGA database. **(I–L)** Scatter plots showing the correlations of CYR61 expression with infiltrated resting memory CD4 T cells **(I)**, naive B cells **(J)**, M2 type macrophages **(K)** and Treg cells **(L)** in ER+ BRCA samples in the TCGA database.

To further explore the role of CYR61 in BRCA, we next assessed whether CYR61 expression is associated with remodeling of the TIME using the TCGA data. As shown in **Figures 4A, B**, CYR61 expression positively correlates with stromal scores, immune scores and estimate scores, either in BRCA or in ER-positive BRCA. Among the 22 types of immune cells investigated, the results of violin plots showed that lower CYR61 expression correlated with reduced infiltrations of antitumor immune cells in both BRCA and ER-positive BRCA patients, including resting memory CD4 T cells and naive B cells (**Figures 4C, D**). On the other hand, CYR61 expression was negatively correlated with the infiltration of some protumor immune cell types, such as Treg cells and M2 type macrophages (**Figures 4C, D**). In addition, scatter plot analyses also confirmed the relationships between CYR61 expression and the infiltrations of these immune cells in both BRCA and ER-positive BRCA tissues (**Figures 4E–L**). These results suggested that downregulation of CYR61 is associated with an impaired antitumor immune microenvironment in BRCA, potentially contributing to tumor progression and immune evasion.

### 3.4 CYR61 expression relates to tumor-suppressive genes and pathways in ER-positive breast cancer

**Figure 5:**
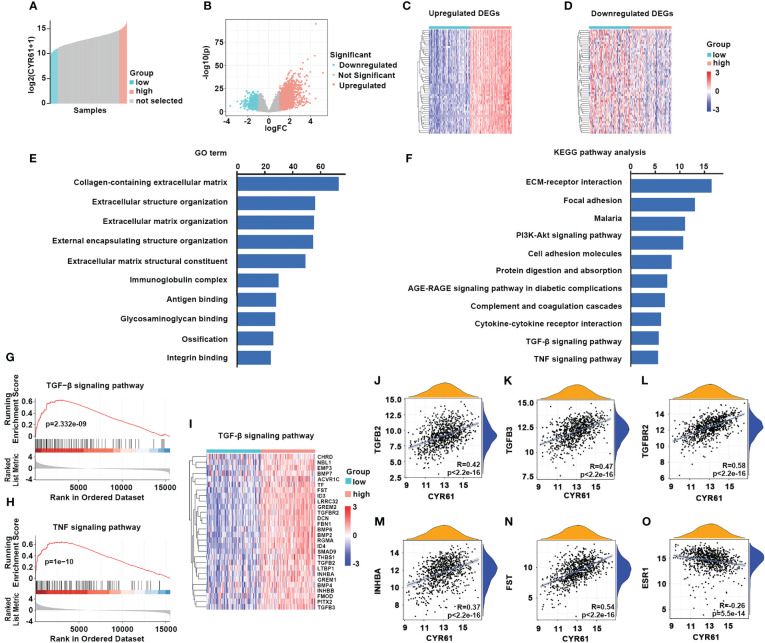
Analyses of CYR61-associated genes and pathways in ER-positive BRCA. **(A)** Comparison of 10% highest and 10% lowest CYR61 expression patients of ER+ BRCA from TCGA database. **(B)** Volcano plot showing the DEGs associated with CYR61 expression in ER+BRCA patients. **(C, D)** Heatmaps showing the DEGs that were positively **(C)** or negatively **(D)** associated with CYR61 expression. **(E, F)** GO term **(E)** and KEGG pathway **(F)** enrichment analyses of CYR61 DEGs. **(G, H)** GSEA analyses of the relationships of CYR61 DEGs to gene sets of TGF-β signaling **(G)** or TNF signaling **(H)**. **(I)** Heatmap for CYR61 DEGs linked to the TGF-β signaling pathway. **(J–O)** Scatter plots displaying the correlations of CYR61 expression to the expression levels of some typical TGF-β pathway genes **(J–N)** or to that of ESR1 **(O)**.

Next, we investigated the possible mechanisms of CYR61 in BRCA. To this end, ER-positive BRCA patients with 10 percent highest and 10 percent lowest CYR61 expression were compared (**Figure 5A**). Using |log_2_(fold change)| >1.0, p <0.05, and count value >30 as the cutoff, 2408 differentially expressed genes (DEGs) were identified between the two groups, including 1629 upregulated genes and 779 downregulated genes) (**Figure 5B**). The top 50 genes that were positively or negatively correlated with CYR61 expression level were shown in heatmaps (**Figures 5C, D**). GO term and KEGG pathway enrichment analyses indicated that these DEGs were closely associated with extracellular matrix (ECM) composition and organization, and regulation of cell adhesion or integrin signaling (**Figures 5E, F**), which is consistent with the typical role of CYR61 as a matricellular protein. In addition, as shown in **Figures 5E, F**, the CYR61 DEGs were also related to the TGF-β and TNF signaling pathways, which have been reported to suppress the growth of ER-positive BRCA (17, 18), as well as genes related to cytokine-receptor interactions, which are crucial for remodeling the TIME (19). GSEA analyses showed that CYR61 DEGs were positively correlated with TGF-β and TNF signaling pathway genes (**Figures 5G–I**). Finally, scatter plot results showed that CYR61 expression displayed a positive correlation with the TGF-β pathway genes, such as TGFB2, TGFB3, TGFBR2, INHBA and FST in ER-positive BRCA (**Figures 5J–N**). However, the expression of CYR61 was negatively associated with ESR1 which encodes estrogen receptor alpha (**Figure 5O**).

### 3.6 CYR61 inhibits the proliferation and colony formation of ER-positive breast cancer cells

**Figure 6:**
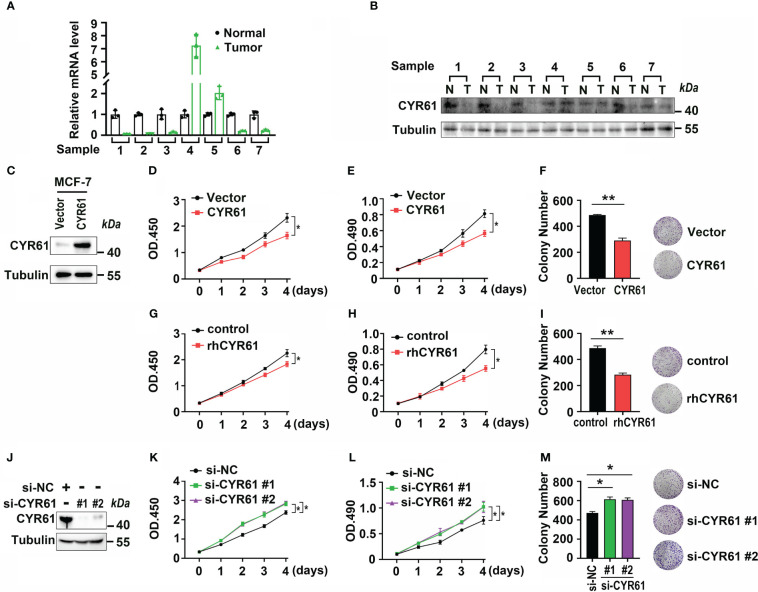
CYR61 inhibits the proliferation and colony formation of ER-positive BRCA cells. **(A, B)** q-PCR **(A)** and western blotting **(B)** analyses of CYR61 expression in 7 ER+ BRCA tissues and their matched paracancer normal breast tissues from patients. **(C)** Verification of CYR61- or the control vector-overexpressing MCF-7 stable cells by western blotting. **(D–F)** MCF-7 cells overexpressing CYR61 or the control vector were subjected to cell proliferation **(D, E)** and colony formation **(F)** examinations. **(G–I)** MCF-7 cells were treated with or without 10 ng/ml recombinant human CYR61 protein (rhCYR61) for the indicated time periods, followed by cell proliferation **(D, E)** and colony formation **(F)** examinations. **(J–M)** MCF-7 cells were transfected with a non-specific (NS) or CYR61-targeting siRNAs for 36 h, followed by western blotting **(J)**, cell proliferation **(K, L)** and colony formation **(M)** assays. *p<0.05; **p<0.01.

The above results suggest that downregulation of CYR61 is an important factor contributing to the pathogenesis of BRCA, especially ER-positive BRCA. Subsequently, we verified the expression and functions of CYR61 experimentally. To support our bioinformatic data, the expression of CYR61 was dramatically downregulated in 5 out of 7 clinically obtained ER-positive BRCA tissues compared to matched paracancer normal breast tissues, at either the mRNA or protein level (**Figures 6A, B**). Then we established MCF-7-derived stable cell lines overexpressing CYR61 or the control vector (**Figure 6C**). Indeed, MTT, CCK8 and colony formation assays showed that ectopic CYR61 was able to attenuate the proliferation and growth of MCF-7 cells (**Figures 6D–F**). Similar results were obtained when MCF-7 cells were administered with recombinant human CYR61 protein (rhCYR61) (**Figures 6G–I**). Another piece of evidence was that depletion of CYR61 expression in MCF-7 cells via siRNAs enhanced the proliferation and colony formation of cancer cells (**Figures 6J–M**).

## 4 Discussion

In the present study, we firstly integrated the data from the TCGA and GTNx cancer databases and performed a pan-cancer analysis of CYR61 expression. CYR61 was found to be downregulated in the majority of the 22 cancer types, such as breast cancer (BRCA), adrenocortical cancer (ACC), bladder cancer (BLCA), and cervical cancer (CESC), whereas upregulated in glioblastoma (GBM), pancreatic carcinoma (PAAD), large B-cell lymphoma (DLBC), and thymoma (THYM) (**Figure 1A**). This is in accordance with previous studies showing that CYR61 is aberrantly expressed in several cancers (9, 10, 20). In addition, CYR61 has been reported to either promote or suppress cancer progression, depending on cancer types (9, 10). However, the expressional alteration and functional role of CYR61 in BRCA are less understood and controversial. While some studies implicated that CYR61 is upregulated in BRCA, one notable study reported that the expression of CYR61 is conversely associated with that of ESR1, which encodes the major estrogen receptor (13, 21–23). Furthermore, both CYR61 and ESR1 genes are regulated by Hippo/YAP signaling in BRCA. YAP signaling suppresses the development of ER-positive BRCA by inhibiting ESR1 gene expression, while simultaneously induces CYR61 expression (13, 14). Our study using updated datasets in the TCGA revealed that CYR61 expression is downregulated in different subtypes or developing stages of BRCA tissues when compared with normal breast tissues (**Figure 1**). Moreover, we also analyzed the methylation status of CYR61 gene promoter based on the TCGA-BRCA database. Intriguingly, reduced expression of CYR61 is well correlated with elevated methylation level of its promoter (**Figure 1**). These results strongly suggest that CYR61 expression in BRCA is not only controlled by upstream signaling pathways, but also influenced by epigenetic mechanisms.

The current classification of BRCA distinguishes three main subgroups, including hormone receptor (ER and/or PR)-positive/HER2-negative (HR+/HER2-), HER2-positive (HER2+, amplified/overexpressed) and triple-negative (ER-/PR-/HER2-) subtypes (3, 24). With the highest incidence in women, BRCA is a major health challenge globally (3). As such, different therapeutic strategies have been developed to cope with BRCA, such as hormone/endocrine therapy for HR-positive/HER2-negative BRCA, chemotherapy and targeted therapy for HER2-positive and triple-negative BRCA, in addition to surgery resection and radiotherapy (24–26). However, drug resistance usually occurs and causes cancer recurrence and relapse (3, 24). Thereafter, there is an urgent need to identify novel prognostic markers and therapeutic targets. Our results indicated that, based on the TCGA and METABRIC databases, lower CYR61 expression levels were associated with OS and DSS rates in all BRCA or ER-positive BRCA patients (**Figure 3**), implicating that CYR61 may serve as a new prognostic marker in BRCA.

BRCA progression depends not only on intrinsic cues in cancer cells, but also on the tumor microenvironment (TME) especially the tumor immune microenvironment (TIME), which provides a proper soil for cancer cell survival, proliferation and dissemination (7). We have compared the immune cell infiltrations between CYR61-low and -high BRCA patients using TCGA data (**Figure 4**). As a result, in both BRCA and ER-positive BRCA, the CYR61 expression level positively correlated with the infiltrations of some antitumor immune cell types including resting memory CD4 T cells and naive B cells, whereas negatively correlated with the infiltrations of Treg cells and M2 type macrophages that play a crucial role in immunosuppression. These results suggest that downregulation of CYR61 in BRCA is associated with a TIME of attenuated antitumor immunity, thereby favoring cancer progression. Our results are also in accordance with previous studies showing that CYR61 could either positively or negatively regulates the inflammatory responses in the liver and cancer immunity in pancreatic adenocarcinoma, in a context-dependent manner (27–29). In addition, in consideration that the single-cell and spatial RNA sequencing approaches have displayed a powerful capability and been widely used in elaborating the composition and function of TME/TIME (30–32), it is important to further explore the role of CYR61 in the regulation of BRCA immunity by utilizing single-cell and spatial omics approaches in the future.

Given that ER-positive BRCA is the most prevalent subtype, we further explored the underlying mechanisms and functional actions of CYR61 in ER-positive BRCA. By comparing high and low CYR61 expression patients, 2408 DEGs were found to be relevantly expressed with the CYR61 expression level (**Figure 5**). GO term and KEGG pathway analyses showed that these DEGs were associated with the known roles of CYR61 in regulating cell matrix formation and organization, ECM-receptor interaction, cell adhesion and integrin signaling. In addition, the CYR61 DEGs are also linked to some tumor-suppressive pathways, including TGF-β and TNF signaling, and also to gene sets of cytokine-cytokine receptor interaction, which may play a role in remodeling the TIME (17–19). Finally, experimental assays confirmed that CYR61 is downregulated in most of the clinic ER-positive BRCA tissues and acts as an inhibitor of cancer cell proliferation and colony formation (**Figure 6**).

In summary, by combining bioinformatic and experimental studies, we found that reduced expression level of CYR61 may serve as a new prognostic marker for ER-positive BRCA, and revealed that CYR61 acts as a tumor inhibitor by impeding cancer cell malignant transformation and remodeling the TIME.

## Data availability statement

The original contributions presented in the study are included in the article/supplementary material. Further inquiries can be directed to the corresponding authors.

## Ethics statement

The studies involving humans were approved by the Medical Ethics Committee of Third Hospital of Nanchang. The studies were conducted in accordance with the local legislation and institutional requirements. The participants provided their written informed consent to participate in this study.

## Author contributions

CZ: Data curation, Formal analysis, Funding acquisition, Investigation, Writing – original draft, Methodology. ZL: Formal analysis, Funding acquisition, Investigation, Methodology, Resources, Writing – original draft. KH: Data curation, Investigation, Methodology, Software, Visualization, Writing – original draft. YR: Data curation, Investigation, Methodology, Software, Writing – original draft. HZ: Data curation, Investigation, Visualization, Writing – original draft. YZ: Data curation, Methodology, Software, Writing – original draft. WW: Data curation, Methodology, Software, Writing – original draft. ST: Funding acquisition, Methodology, Supervision, Writing – review & editing, Conceptualization. XY: Conceptualization, Funding acquisition, Supervision, Writing – review & editing.
